# Real-world analysis of genotype-guided treatment for carriers of actionable *DPYD* and *UGT1A1* variants

**DOI:** 10.1038/s41397-026-00432-2

**Published:** 2026-10-12

**Authors:** Amy L. Pasternak, William Tackett, Mousumi Banerjee, Simi Paul, Emily Bertucci-Richter, Xiang Zhou, Vaibhav Sahai, Daniel L. Hertz

**Affiliations:** 1 Department of Clinical Pharmacy, University of Michigan College of Pharmacy, Ann Arbor, MI USA; 2 Department of Pharmacy, University of Michigan Health, Ann Arbor, MI USA; 3 Department of Biostatistics, University of Michigan School of Public Health, Ann Arbor, MI USA; 4 Division of Hematology and Oncology, Department of Internal Medicine, University of Michigan Medical School, Ann Arbor, MI USA

**Keywords:** Cancer genetics, Genetic association study, Genetics research, Genetic testing, Drug safety

## Abstract

This retrospective, matched, case-control study was conducted to compare rates of severe adverse events (SAE) within the first three treatment cycles in *DPYD* and *UGT1A1* variant carriers who received genotype-guided treatment compared to variant carriers who received standard treatment with fluoropyrimidines (FP) and irinotecan. Subjects were matched by at-risk phenotype, chemotherapy regimen, and age. Sixteen subjects received genotype-guided treatment (12 with FP) and were matched to 75 subjects who received standard treatment. The primary endpoint of SAE attributed to the FP or irinotecan was significantly lower in variant carriers receiving genotype-guided treatment (12.5% vs. 50.7%; OR: 0.15; 95% CI: 0.03–0.68). Treatment modifications were also significantly reduced for genotype-guided treatment (12.5% vs. 54.6%; *p* < 0.05). Six (37.5%) subjects with genotype-guided treatment received and tolerated dose escalation. This real-world study of genotype-guided treatment in *DPYD* and *UGT1A1* variant carriers provides additional evidence of the clinical benefit of genotype-guided treatment to improve patient safety.

## Introduction

The fluoropyrimidines (FP), 5-fluorouracil (5-FU) and capecitabine, and irinotecan are commonly utilized chemotherapies with known pharmacogenetic interactions. FPs undergo metabolism by dihydropyrimidine dehydrogenase (DPD); genetic variation in the *DPYD* gene can cause reductions in DPD activity and significantly increase the risk of severe, potentially life-threatening adverse events to standard FP therapy [1]. Irinotecan’s active metabolite, SN-38, is dependent on UDP-glucuronosyltransferase 1A1 (UGT1A1) activity for elimination. Genetic variation in the *UGT1A1* gene can reduce UGT1A1 enzyme activity and increase the risk of severe adverse events to standard irinotecan treatment [2]. Both medications have clinical practice guideline recommendations from multiple organizations that recommend adjusting the initial FP or irinotecan dose based on the patient’s *DPYD* or *UGT1A1* genotype, respectively [1–5].

Despite the well-established relationship between the pharmacogenetic variants and toxicity risk and the availability of recommendations for dose modification, there has historically been limited utilization of pharmacogenetic testing in patients receiving FP or irinotecan in the United States. In studies conducted before FDA labeling changes to recommend *DPYD* testing prior to FP use, only 20% of surveyed providers indicated routinely performing preemptive *DPYD* testing for FP, with only 1% reporting preemptive *UGT1A1* testing for irinotecan [6, 7]. Lack of evidence of the impact of genotype testing on clinical outcomes and lack of a clinical practice guideline recommendation to perform pre-treatment testing, which subsequently impacts insurance coverage, were commonly identified barriers to utilization [6, 8].

The purpose of this study was to assess the effect of genotype-guided FP and irinotecan treatment on treatment-related SAE and tolerance in a real-world population of *DPYD* and *UGT1A1* variant carriers. It was hypothesized that genotype-guided dosing would improve treatment safety, further demonstrating the clinical benefits of pre-treatment pharmacogenetic testing.

## Methods

### Ethics approval and consent to participate

This study was conducted at a single site in accordance with the Declaration of Helsinki and applicable local regulations. The study protocol was approved by the University of Michigan institutional review board (HUM00213709). A waiver of informed consent was approved within the study protocol.

### Identification of variant carriers with genotype-guided treatment

Patients ≥ 18 years of age who underwent clinical genetic testing between 07/2014-05/2024 for *DPYD* or *UGT1A1* prior to starting FP or irinotecan treatment were evaluated for inclusion. *DPYD* genotyping was conducted at Mayo Clinic Laboratories and assessed eight *DPYD* variants (c.1905+1G > A, c.299_302del, c.703C > T, c.2983G > T, c.1679T > G, c.2846A > T, c.1129-5923C > G, c.557A > G) [9]. Identification of any single variant or combination of the variants was considered a *DPYD* variant carrier for study inclusion. *UGT1A1* genotyping was performed at Michigan Medicine Laboratories and detected the number of tandem TA repeats for the determination of the UGT1A1*36 (5 TA repeats), UGT1A1*28 (7 TA repeats), and UGT1A1*37 (8 TA repeats) alleles; subjects who were homozygous or compound homozygous for the UGT1A1*28 or *37 variants were considered *UGT1A1* variant carriers for inclusion [10].

EHR records were reviewed for documentation that the initial FP- or irinotecan dose was adjusted by the medical oncologist because of the identified *DPYD* or *UGT1A1* variant. As dose adjustment recommendations from clinical guidelines varied over the study time frame, and medical oncologists may have accounted for other non-genetic factors in dosing decisions, all instances where the initial dose reduction was attributed to the identified genotype in the EHR were considered genotype-guided treatment [1, 11]. Subjects were excluded if they had a prior allogeneic stem cell or liver transplant or had previously received treatment with FP or irinotecan prior to the clinical genotyping.

### Identification of variant carriers with standard treatment

Patients ≥ 18 years of age who received FP or irinotecan between 07/2014-05/2024 without clinical *DPYD* or *UGT1A1* genotype testing but who were enrolled in the Michigan Genomics Initiative (MGI) institutional genetic data repository were assessed for inclusion [12, 13]. The same *DPYD* variants as the clinical genotype test were evaluated in MGI to identify *DPYD* variant carriers who received standard FP treatment. The *UGT1A1* *80/*80 genotype was evaluated as the *UGT1A1* TATA repeat variants were not detectable. However, as the UGT1A1*80 allele is in very high linkage disequilibrium with UGT1A1*28 and UGT1A1*37, clinical guidelines recommend detection can be used to infer decreased function when it is the only allele tested [14]. Subjects who received standard treatment were also excluded for prior history of allogeneic stem cell or liver transplant or if they had previously received genotype-guided FP or irinotecan dosing.

Variant carriers who received standard treatment were then matched with the variant carriers in the genotype-guided group by DPD or UGT1A1 phenotype, chemotherapy regimen (i.e., chemotherapy agent and monotherapy or combination therapy, Supplementary Table 1), and age at time of treatment (+/− 10 years). For matching, the patient’s DPD phenotype was classified as an intermediate metabolizer (IM) or poor metabolizer (PM) according to the Clinical Pharmacogenetics Implementation Consortium (CPIC) DPD activity score (Supplementary Table S2) [1]. Patients with homozygous or compound homozygous *UGT1A1* variant genotypes were considered UGT1A1 PM per CPIC definitions [14].

### Data collection and outcomes

The primary outcome was the rate of grade 3 or higher adverse events (grade 3 + AE) or SAE attributable to FP or irinotecan during the first three cycles of treatment. Adverse events were assessed via retrospective chart review of laboratory tests and clinical notes and graded according to the Common Terminology Criteria for Adverse Events (CTCAE) Version 5.0 [15]. SAE were defined as any event that was life-threatening, resulted in hospitalization or prolongation of existing hospitalization for >24 h, or required additional medical interventions to prevent progression to an event requiring the above. The attributed cause of all grade 3 + AE or SAE according to the medical oncology team was also collected from clinical notes. If the grade 3 + AE or SAE was not specifically related to a different etiology by the treating medical oncology team, the toxicity was maintained as attributable to the FP or irinotecan in the analysis. As clinical genetic data was documented within the EHR, subject group assignment was not blinded. Secondary outcomes included the rate of treatment alterations of FP or irinotecan that were attributed to FP- or irinotecan associated toxicity by the treating clinician in the EHR. Treatment alterations included dose reductions ( > 10%), treatment delays, defined as any prolongation in the scheduled treatment cycle, or early treatment discontinuation. Dose escalations within the first three treatment cycles were also collected and were defined as a > 10% increase in the chemotherapy dose administered compared with the initial cycle.

### Statistical analysis

All included subjects that completed at least one chemotherapy cycle were included in the analysis. Demographics were reported using descriptive statistics. Differences between variant carriers who received genotype-guided versus standard treatment were evaluated using t-tests for continuous data or chi- squared tests for categorical data. Fisher’s exact test was used when the expected cell frequency in a contingency table was less than 5. The primary analysis compared the rate of grade 3 + AE or SAE attributed to FP or irinotecan between matched subjects who received genotype-guided treatment versus standard treatment via univariate conditional logistic regression, resulting in odds ratios adjusted for the matched design. The primary outcome was also compared between the matched subjects stratified by chemotherapy (e.g., FP or irinotecan). Conditional logistic regression was used to model the likelihood of grade 3 + AE or SAE as a function of genotype-guided treatment, sex, cancer type (i.e., gastrointestinal/pancreatic vs. breast vs. other), DPD activity score, and chemotherapy regimen (monotherapy vs dual agent [e.g. FOLFOX] vs triple agent therapy [e.g. FOLFIRNOX]) for *DPYD*-FP subjects. Chemotherapy treatment alterations (dose reduction, treatment delay, or discontinuation) were also compared between the matched subjects via univariate conditional logistic regression. Dose escalation data were reported descriptively.

## Results

### Subject screening and enrollment

A total of 91 subjects were included in this study, including 16 patients with genotype-guided treatment and 75 matched subjects with standard treatment (median (range) of 4.5 (1–9) standard treatment subjects matched to each genotype-guided subject). Demographics of the included patients are summarized in Table 1. Twelve (75%) were DPD IM who received genotype-guided FP and were matched with 56 DPD IM (74.7%) patients with standard treatment, while four (25%) were UGT1A1 PM who received genotype-guided irinotecan and were matched to 19 (25.3%) UGT1A1 PM subjects with standard treatment. The identified genotypes for *DPYD* variant carriers are listed in Supplementary Table 2. No subjects experienced an interaction for both *DPYD*-FP and *UGT1A1*-irinotecan. The most common cancer diagnosis for genotype-guided treatment was rectal cancer (31.2%) while pancreatic cancer (25.7%) was the most common in standard treatment, although this difference was not significant. There were no significant differences among any other clinical variables between the groups. Initial doses of 5-FU, capecitabine, and irinotecan were reduced by a median of approximately 50%, 30%, and 40%, respectively, for subjects with genotype-guided treatment, whereas all standard treatment subjects initiated treatment at standard dose (i.e., 100%).

**Table 1:** Demographic and Clinical Data of Included *DPYD* or *UGT1A1* Variant Carriers (n = 91).

		Genotype-guided treatment (*n* = 16)	Standard treatment (*n* = 75)	*p*-value
**Self-reported Race**	White	16 (100%)	67 (89.3)	0.6
	Black	0 (0%)	5 (6.7%)	
	Other	0 (0%)	3 (4.0%)	
**Sex**	Male	9 (56.2%)	32 (42.7%)	0.48
**Genetics Phenotype**	DPD IM (AS = 1.5)	9(56.2%)	47 (62.7%)	0.75
	DPD IM (AS = 1.0)	3 (18.8%)	9 (12.0%)	
	DPD PM	0	0	
	UGT1A1 PM	4 (25.0%)	19 (25.3%)	
**Cancer Type**	Colorectal	10 (62.4%)	27 (36.0%)	0.48
	Pancreatic	2 (12.5%)	19 (25.7%)	
	Breast	3 (18.8%)	12 (16.2%)	
	Gastric	1 (6.2%)	2 (2.7%)	
	Other	0 (0%)	14 (18.7%)	
**Chemotherapy Regimen**	5-FU combination therapy (no irinotecan)	5 (31.2%)	23 (31.1%)	0.94
	Capecitabine only	4 (25%)	19 (25.7%)	
	Capecitabine combination therapy (no irinotecan)	2 (12.5%)	4 (5.4%)	
	FOLFIRI or FOLFIRINOX or other irinotecan combination	5 (31.3%)	18 (24%)	

*IM* Intermediate metabolizer, *PM* Poor Metabolizer, *5-FU* 5-Fluorouracil, *AS* Activity Score.

### Comparison of AEs between genotype-guided and standard treatment

**Fig. 1:**
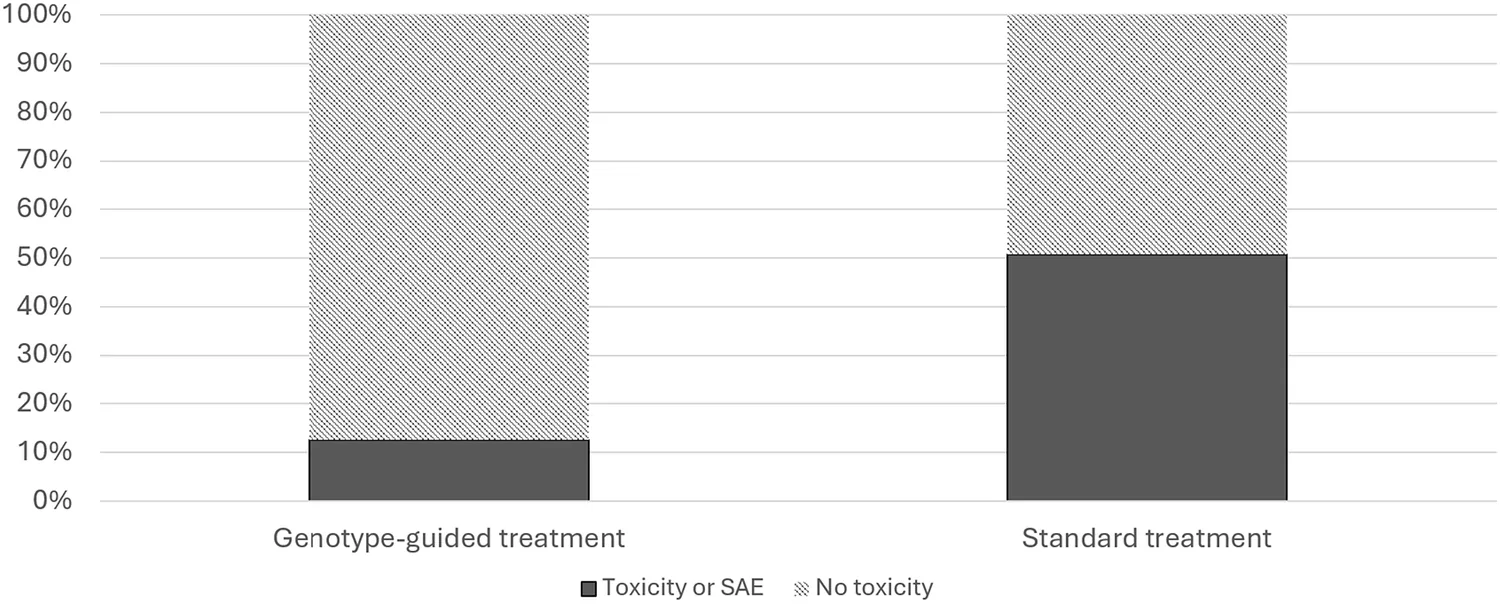
Percentage of subjects with Toxicity Attributable to Index Drug (FP or irinotecan) in the genotype-guided versus standard treatment groups. There was a significantly lower rate of toxicity attributable to treatment in the genotype-guided group (12.5% vs. 50.7%, OR = 0.15, 95% CI: 0.03–0.68, *p* < 0.05).

In the primary analysis, the proportion of patients who experienced a grade 3 + AE or SAE attributed to FP or irinotecan during the first three treatment cycles was lower in the variant carriers with genotype-guided treatment compared to standard treatment (12.5% vs. 50.7%, OR: 0.15; 95% confidence interval [95% CI]: 0.03–0.68, Table 2, Fig. 1) overall and in variant carriers receiving genotype-guided FP (Table 2). None of the additional variables including sex, cancer type, DPD activity score, or regimen type were associated with toxicity risk in the conditional logistic regression model (data not shown). The incidence of any treatment alteration (i.e., dose decrease, delay, or discontinuation) was significantly lower in the genotype-guided treatment than standard treatment group (8.0% vs. 54.6%, *p* < 0.01, Table 2). The observed rate of the primary and secondary outcomes by observed *DPYD* variant are provided in Supplementary Table 2.

**Table 2:** Toxicity Outcomes among *DPYD* or *UGT1A1* Variant Carriers receiving Genotype-guided or Standard Dosing within the first three treatment cycles.

	Genotype-guided (n/N)	Standard treatment (n/N)	OR (95% CI)	*p*-value
*Grade 3* + *AE or SAE attributable to FP or irinotecan treatment*				
**All patients**	12.5% (2/16)	50.7% (38/75)	0.15 (0.03–0.68)	0.02
**FP-only**	8.3% (1/12)	50.0% (28/56)	0.09 (0.01–0.74)	0.03
**Irinotecan-only**	25% (1/4)	52.6% (10/19)	0.36 (0.04, 3.61)	0.38
*Treatment alterations*				
**All patients**	8.0% (2/16)	54.6%% (41/75)	0.12 (0.03–0.56)	<0.01

*AE* adverse event, *SAE* Severe adverse event, *FP* fluoropyrimidine.

Of the 16 patients who received genotype-guided treatment, six (37.5%) had a dose escalation for their second or third treatment cycle. Five were DPD IMs who initiated FP at ~50% of the standard starting dose (Supplementary Table 3). All five patients tolerated the received dose escalation, which ranged from 62–100% of the standard dose. Additionally, one UGT1A1 PM who initiated irinotecan with a 50% dose reduction tolerated an increase to 75% of the standard dose.

## Discussion

*DPYD* and *UGT1A1* testing and pharmacogenetics-guided chemotherapy dosing are increasingly being used to prevent severe treatment-related adverse effects. This study provides further evidence of the clinical benefit of this strategy, including a reduction of adverse effects attributable to chemotherapy and a reduction of treatment alteration due to toxicity.

These results are consistent with other studies that have evaluated toxicity in *DPYD* or *UGT1A1* variant carriers receiving genotype-guided dosing. PREPARE was a prospective trial in which patients were randomized to panel PGx genotyping or standard care without genotyping, but who were retrospectively genotyped for analysis [16]. A secondary analysis of genotype-guided FP (50% reduction) and irinotecan (30% reduction) dosing in patients with gastrointestinal cancer found that this strategy significantly reduced the rate of toxicities (OR: 0.1; 95% CI: 0.0–0.8) [17]. We performed a post-hoc power calculation based on Monte-Carlo simulations comparing our findings to the results of the PREPARE study. Based on the simulations, our study design had 85% power to detect a statistically significant difference in SAEs between the genotype-guided and standard treatment groups at alpha=0.05. Two other single-arm trials (IMPACT-GI [18] and PACIFIC-PGx [19]) prospectively genotyped *DPYD* and *UGT1A1* in patients initiating FP or irinotecan treatment and compared toxicity rates for variant carriers receiving genotype-guided treatment vs. retrospectively identified variant carriers who had received standard doses. These studies also reported that initial dose reduction reduces SAE and treatment modifications.

Although this study confirms the benefits of genotype-guided dose reductions on treatment safety, it also highlights the need for dose escalation, especially for FP. Patients carrying *DPYD* c.1236 G > A (i.e., HapB3) who receive FP-dose reductions may have reduced progression-free survival compared with wild-type patients receiving standard dosing (HR, 1.43; 95% CI, 1.10 to 1.86; *P* = 0.007) [20]. *DPYD* variant carriers receiving genotype-guided dose reductions have been observed to have lower systemic FP concentrations compared to wild-type patients receiving standard dosing [21]. These data suggest that without the incorporation of the recommended FP dose escalation [1, 22], there is potential under-treatment from genotype-guided FP-dosing. Conversely, studies have reported that systemic concentrations of SN-38 remain elevated in UGT1A1 PM who receive a 30% irinotecan dose reduction [23, 24]. No studies have reported a difference in clinical outcomes, although studies that have evaluated this are limited [25, 26] so the impact of genotype-guided dosing for irinotecan on clinical response warrants further study.

Additionally, the previous studies evaluating treatment efficacy have not compared outcomes for variant carriers receiving reduced doses vs. variant carriers receiving standard doses, which is the primary comparison of interest. The secondary analysis of PREPARE found that initial dose reduction increased the overall dose intensity that can be administered in *DPYD* variant carriers, suggesting this strategy may improve efficacy [17]. It is likely infeasible to definitively determine the effect of initial dose reduction on efficacy in a prospective clinical trial, but ongoing pooled analyses of real-world data could provide useful evidence [27] to fully understand the clinical benefits and risks of initial genotype-guided dose reduction.

Regardless of the unanswered questions about clinical risks, there is general consensus that *DPYD* genotyping and genotype-guided FP dosing should be used in clinical practice to prevent severe and fatal toxicity [28–30]. Our study, and other similar studies, demonstrate the feasibility and suggest a potential added benefit of including *UGT1A1* testing in patients who will also receive irinotecan. Although only a small number of subjects received *UGT1A1*-guided irinotecan in this study, limiting definitive conclusions, the findings align with a larger, real-world analysis that observed an approximately 50% reduction in toxicity in UGT1A1 PM receiving genotype-guided dosing compared to UGT1A1 PM who received standard dosing [31]. Testing for both *DPYD* and *UGT1A1* could help discern the risk of toxicity for multiple components of the patient’s treatment regimen or identify patients with enhanced toxicity risk secondary to altered metabolism for both FP and irinotecan.

Additional benefits could also be realized through more comprehensive pharmacogenetic testing. It has been estimated that up to one-quarter of patients with cancer receive at least one medication where they carry a genetic variant with a corresponding pharmacogenetic treatment recommendation; in many instance that medication is not a chemotherapy [16, 32, 33]. For example, pharmacogenetic interactions for opioids commonly prescribed for cancer-related pain may contribute to increased hospital encounters [34]. A comprehensive pharmacogenetic testing approach could therefore help to improve treatment safety and efficacy across cancer-directed and supportive treatments.

Limitations of the current study should be considered. Not all known actionable variants were evaluated on the clinical testing assays (i.e. UGT1A1*6), so it is possible that patients with increased toxicity risk could have been missed; however as only patients with homozygous or compound homozygous *UGT1A1* variants or actionable *DPYD* variants were included, a missed variant would have resulted in exclusion from the study and would not be anticipated to significantly impact the analysis. The retrospective unblinded data collection could have led to observer bias during data collection between the genotype-guided and standard treatment subjects, which we attempted to minimize through matching. As clinical testing was performed at the discretion of the medical oncologist, there may also be potential bias related to selection of patients for genotype-guided dosing which could also impact toxicity monitoring and attribution. Although all subjects were identified within the same timeframe, there is the potential for variability in clinical practice and possible temporal bias between those who received genotype-guided versus standard treatment. The retrospective design also introduces risk for incomplete data collection and use of supportive therapeutics such as granulocyte-colony stimulating factors were not evaluated. The modestly sized cohort precluded matching by the specific genetic variation identified for *DPYD*; although it is known different variants have different impact on DPD activity, however the observed genotype-guided dose reductions were similar for the same variants across the genotype-guided cohort. The cohort size also limited power to control for other characteristics that contribute to toxicity risk, such as performance status, body mass index, or other comorbidities and to conduct subpopulation analyses to assess toxicity reductions for *UGT1A1*-irinotecan.

In conclusion, this real-world study adds to the growing evidence of the feasibility and clinical benefits of pre-treatment genotyping of *DPYD* and *UGT1A1* and genetics-informed dosing for patients initiating FP and/or irinotecan treatment. It also highlights the importance of considering dose-escalation for DPD IM’s who receive initial FP dose-reductions. Future work is needed to increase clinical uptake of this strategy and collect real-world data to demonstrate the clinical benefits of pharmacogenetic testing to reduce toxicity and improve outcomes in patients with cancer.

## Supplementary information

Supplementary Table 1

Supplementary Table 2

Supplementary Table 3
